# Profile of epigenetic mechanisms in lung tumors of patients with underlying chronic respiratory conditions

**DOI:** 10.1186/s13148-017-0437-0

**Published:** 2018-01-16

**Authors:** Mercè Mateu-Jimenez, Víctor Curull, Alberto Rodríguez-Fuster, Rafael Aguiló, Albert Sánchez-Font, Lara Pijuan, Joaquim Gea, Esther Barreiro

**Affiliations:** 1 Pulmonology Department, Lung Cancer and Muscle Research Group, Hospital del Mar-IMIM, Parc de Salut Mar, Health and Experimental Sciences Department (CEXS), Universitat Pompeu Fabra (UPF), Parc de Recerca Biomèdica de Barcelona (PRBB), C/ Dr. Aiguader, 88, E-08003 Barcelona, Spain; 2 Centro de Investigación en Red de Enfermedades Respiratorias (CIBERES), Instituto de Salud Carlos III (ISCIII), Barcelona, Spain; 3 Thoracic Surgery Department, Hospital del Mar-IMIM, Parc de Salut Mar, Barcelona, Spain; 4 Pathology Department, Hospital del Mar-IMIM, Parc de Salut Mar, Barcelona, Spain

**Keywords:** Increased susceptibility to lung cancer, Underlying chronic respiratory disease, Tumor microRNAs, DNA methylation, Lung tumorigenesis biomarkers

## Abstract

**Background:**

Chronic lung diseases such as chronic obstructive pulmonary disease (COPD) and epigenetic events underlie lung cancer (LC) development. The study objective was that lung tumor expression levels of specific microRNAs and their downstream biomarkers may be differentially regulated in patients with and without COPD.

**Methods:**

In lung specimens (tumor and non-tumor), microRNAs known to be involved in lung tumorigenesis (miR-21, miR-200b, miR-126, miR-451, miR-210, miR-let7c, miR-30a-30p, miR-155 and miR-let7a, qRT-PCR), DNA methylation, and downstream biomarkers were determined (qRT-PCR and immunoblotting) in 40 patients with LC (prospective study, subdivided into LC-COPD and LC, *N* = 20/group).

**Results:**

Expression of miR-21, miR-200b, miR-210, and miR-let7c and DNA methylation were greater in lung tumor specimens of LC-COPD than of LC patients. Expression of downstream markers *PTEN*, *MARCKs*, *TPM-1*, *PDCD4*, *SPRY-2*, *ETS-1*, *ZEB-2*, *FGFRL-1*, *EFNA-3*, and *k-RAS* together with *P53* were selectively downregulated in tumor samples of LC-COPD patients. In these patients, tumor expression of miR-126 and miR-451 and that of the biomarkers *PTEN*, *MARCKs*, *FGFRL-1*, *SNAIL-1*, *P63*, and *k-RAS* were reduced.

**Conclusions:**

Biomarkers of mechanisms involved in tumor growth, angiogenesis, migration, and apoptosis were differentially expressed in tumors of patients with underlying respiratory disease. These findings shed light into the underlying biology of the reported greater risk to develop LC seen in patients with chronic respiratory conditions. The presence of an underlying respiratory disease should be identified in all patients with LC as the differential biological profile may help determine tumor progression and the therapeutic response. Additionally, epigenetic events offer a niche for pharmacological therapeutic targets.

**Electronic supplementary material:**

The online version of this article (10.1186/s13148-017-0437-0) contains supplementary material, which is available to authorized users.

## Background

Lung cancer (LC) continues to be the most common cause of cancer-related mortality worldwide, rising up to one third of all-cause mortality in certain regions [1]. Major respiratory conditions such as chronic obstructive pulmonary disease (COPD) have been consistently proposed as risk factors of LC development in patients [2–7]. Specifically, the presence of emphysema was demonstrated to be the most important risk factor in LC pathogenesis [2–7]. Despite the relevance of this problem, identification of the underlying biology that predisposes patients with airway obstruction and emphysema to LC remains at its infancy.

Several mechanisms such as greater levels of oxidative stress [8–11] and inflammatory markers [8, 12, 13], along with a relative predominance of Th1 cytokines and M1 macrophages, were demonstrated in the tumors and blood of patients with LC and COPD [14]. In patients with underlying chronic respiratory conditions, these biological mechanisms are likely to participate in the neoplastic transformation as they may interfere with important cell processes such as repair, angiogenesis, and apoptosis [8, 12, 13].

Epigenetic events, defined as the process whereby gene expression is regulated by heritable mechanisms that do not alter DNA sequence, significantly contribute to adaptation to environmental factors. In this regard, cigarette smoking-mediated effects were shown to induce epigenetic modifications in the airways and lungs of patients [15–17]. Alterations in several epigenetic mechanisms such as chromatin remodeling, DNA methylation, and either histone acetylation or methylation were shown to occur in the airways of COPD patients and in lung tumors of patients irrespective of COPD [18, 19]. Interestingly, in patients with COPD and LC, methylation of immune-related genes was demonstrated in their tumor samples [20]. In keeping with, several specific immune-related genes were shown to be hypermethylated in tumor samples of patients with underlying COPD, suggesting that methylation of those specific genes may contribute to the epithelial cell transformation to cancer in patients with chronic respiratory conditions [21, 22]. On the other hand, non-coding single-stranded RNA molecules (microRNAs) which regulate cellular processes, namely proliferation, cell invasion and migration, adaptation to hypoxia, apoptosis, and angiogenesis have also been proposed to underlie lung tumorigenesis in patients with chronic respiratory conditions [23–27]. Additionally, specific microRNA expression patterns may also help predict mortality and response to treatment in LC patients [25, 28]. Whether these mechanisms may take place in the actual tumor lesions of LC patients with underlying respiratory conditions remains to be fully explored.

On this basis, we hypothesized that expression levels of specific microRNAs and DNA methylation may be differentially expressed in the lung tumors of patients with underlying respiratory diseases versus those without the presence of chronic respiratory conditions. Moreover, we also hypothesized that expression of a selected panel of biomarkers known to be specifically regulated by the target microRNAs may also differ in the tumors and non-tumor lungs of patients with LC with and without COPD. These downstream biomarkers were carefully chosen on the basis of currently available literature on the biological processes that are regulated by the target LC-associated microRNAs. Hence, the specific study objectives were to assess prospectively in tumor and non-tumor lung specimens of patients with non-small cell LC (NSCLC), with and without underlying COPD: (1) expression levels of microRNAs known to underlie lung tumorigenesis and total DNA methylation and (2) levels of their downstream pathways known to promote cellular processes involved in lung tumorigenesis and progression.

## Methods

### Study patients

This was a prospective, controlled study in which a group of 40 Caucasian patients (33 males, Table 1) with LC, undergoing thoracotomy for their lung neoplasm, were recruited consecutively from the Lung Cancer Clinic of the Respiratory Medicine Department at *Hospital del Mar* (Barcelona, Spain). The patients were always recruited before having received any treatment for their lung neoplasm including chemotherapy and/or radiotherapy. Tumor and non-tumor lung specimens were obtained (*n* = 40), and patients were subdivided into two groups depending on the presence of underlying COPD [26, 27, 29]: (1) 20 patients with LC who also had COPD (LC-COPD group) and (2) 20 patients with LC without COPD (LC group). As part of the prospective ongoing cohort “Lung Cancer-COPD” in our center, both LC and LC-COPD patients simultaneously participated in a previous study aimed to assess Th1 and Th2 inflammatory profiles and their implications in lung tumors of patients with chronic respiratory conditions (14).

**Table 1:** Clinical and functional characteristics of the study patients

Anthropometric variables	Lung cancer *N* = 20	Lung cancer-COPD *N* = 20
Age, years	64 (12)	65 (9)
Male, *N*/female, *N*	13/7	20/0
BMI, kg/m^2^	26 (5)	26 (5)
Smoking history		
Current: *N*, %	12, 60	14, 70
Ex-smoker: *N*, %	5, 25	6, 30
Never smoker: *N*,%	3, 15	0, 0^¶^
Pack-years	53 (25)	57 (20)
Lung function testing		
vFEV_1_, % pred	92 (8)	61 (14)^¶¶¶^
FEV_1_/FVC, % pred	77 (6)	60 (8)^¶¶¶^
DLCO, % pred	88 (12)	72 (21)^¶¶¶^
KCO, % pred	88 (11)	73 (17)^¶¶¶^
TNM staging		
Stage IA: *N*, %	5, 25	5, 25
Stage IB: *N*, %	0, 0	4, 20
Stage IIA: *N*, %	1, 5	6, 30
Stage IIB: *N*, %	4, 20	2, 10
Stage IIIA: *N*, %	3, 15	3, 15
Stage IIIB: *N*, %	4, 20	0, 0
Stage IV: *N*, %	3, 15	0, 0
Histological diagnosis		
Squamous cell carcinoma: *N*, %	6, 30	5, 25
Adenocarcinoma: *N*, %	11, 55	14, 70
Others: *N*, %	3, 15	1, 5
Blood parameters		
Total leucocytes/μL	8.3 10^3^ (1.9 10^3^)	9.2 10^3^ (2.1 10^3^)
Total neutrophils/μL	5.7 10^3^ (2.1 10^3^)	6.3 10^3^ (1.7 10^3^)
Total lymphocytes/μL	1.7 103 (650)	2.1·10^3^ (617)
Albumin (g/dL)	4.4 (0.4)	4.0 (0.5)^¶¶^
Total proteins (g/dL)	7.4 (0.5)	7.1 (0.6)
Fibrinogen (mg/dL)	485 (99)	409 (77)^¶¶^
CRP (mg/dL)	1.1 (1.1)	1.7 (1.2)
GSV (mm/h)	20 (7.4)	30 (16.8)^¶^
Ceruloplasmin (g/dL)	26.2 (4.8)	26.5 (4.8)
Body weight loss, kg		
0, *N*, %	18, 90	17, 85
1–4, *N*, %	2, 10	1, 5
5–8, *N*, %	0, 0	2, 10
9–12, *N*, %	0, 0	0, 0

Continuous variables are presented as mean (standard deviation), while categorical variables are presented as the number of patients in each group and percentage of the total population

*Abbreviations*: *N* number, *kg* kilograms, *m* meters, *BMI* body mass index, *FEV*_*1*_ forced expiratory volume in the first second, *pred* predicted, *FVC* forced vital capacity, *DLco*, carbon monoxide transfer, *K*_*CO*_ Krogh transfer factor, *TNM* tumor, nodes, metastasis, *CRP* C-reactive protein, *GSV* globular sedimentation velocity, *L* liter

^¶^*p* < 0.05, ^¶¶^*p* < 0.01, ^¶¶¶^*p* < 0.001 between LC-COPD and LC patients. Comparisons of the clinical and physiological variables between LC-COPD and LC patients were assessed using the Student’s *t* test. Differences between the study groups in the qualitative variables were assessed using the chi-square test

Approval was obtained from the institutional Ethics Committee on Human Investigation (*Hospital del Mar*–*IMIM*, Barcelona, project number 2014/5776/1) in accordance with the World Medical Association guidelines (Helsinki Declaration of 2008) for research on human beings. Informed written consent was obtained from all patients at study entry and prior to initiation of therapies.

### Anthropometrical and functional assessment

Lung function and anthropometry were evaluated in all patients as previously described [8, 14, 30–32].

### Sample collection

During the surgical intervention (minimal resection or lobectomy), in patients that underwent thoracotomy (*n* = 40), lung samples were obtained (tumor and non-tumor specimens) by the expert lung pathologist. Non-tumor samples were collected from the distant surrounding parenchyma with respect to where the tumor was localized (7-cm minimum distance from the tumor site in all the samples), which enabled us to study non-tumor lung specimens. Briefly, non-tumor samples were carefully selected as a result of visual and manual detection (palpation) of any potential nodules that might modify disease staging. The presence of ground-glass opacities in the non-tumor specimens was always ruled out by previously analyzing the CT scan sections in all the study patients. Additionally, hematoxylin-eosin staining was performed in all non-tumor lung specimens to further confirm the absence of cancer cells. For ethical reasons, this was the only approach approved to study non-tumor lungs in COPD and non-COPD patients in our institution. In all cases, the expert lung pathologist selected a fragment of lung tumor and non-tumor specimens of approximately 10 × 10 mm^2^ size from the fresh samples after a careful collection of the specimens required for diagnosis purposes. On the day of thoracotomy, blood samples were obtained for conventional routine blood tests (hemogram and nutritional parameters). Importantly, a minimum amount of 50% of cancer cells was similarly identified in all tumor types from all the study patients. The remaining cell components were inflammatory and stromal cells in all the analyzed tumors. On the day of thoracotomy, blood samples were obtained for conventional routine blood tests (hemogram and nutritional parameters).

### Sample preservation

Lung specimens (tumor and non-tumor) were snap-frozen in liquid nitrogen and stored at − 80 °C for further use.

### Molecular biology analyses

The biology analyses were all conducted blind in our molecular biology laboratory (IMIM).

#### MicroRNAs and target genes

RNA and DNA isolations and quantitative real time-PCR amplification (qRT-PCR) were used to quantify in the lung specimens, the target microRNAs and downstream biomarkers (genes involved in key cellular processes related to tumorigenesis and cancer progression; Additional file 1: Tables S1 and S2, Tables 2, 3, and 4) of the microRNAs analyzed in the current study [30, 32–34].

**Table 2:** Downstream targets of the microRNAs analyzed in tumor and non-tumor lungs of the study patients

MicroRNAs	Downstream targets	LC patients		LC-COPD patients	
		Non-tumor	Tumor	Non-tumor	Tumor
miR-21					
	*PTEN*, gene expression	78.5 (59.3)	115.6 (89.7)	82.4 (70.2)	0.26 (0.60)^*, §§§^
	*MARCKs*, gene expression	0.4 (0.2)	0.5 (0.2)	0.5 (0.5)	0.2 (0.1)^*, §^
	*TPM-1*, gene expression	2.8 (2.1)	11.0 (10.4)^**^	5.8 (4.9)	1.8 (1.5)^§§^
	*PDCD4*, gene expression	5.4 (3.4)	5.2 (4.4)	4.0 (2.5)	1.4 (1.0)^§^
	*SPRY-2*, gene expression	6.0 (3.3)	8.4 (7.1)	3.4 (1.9)	1.2 (1.0)^§§§^
miR-200b					
	*ETS-1*, gene expression	4.6 (3.7)	3.3 (2.5)	2.8 (2.5)	0.6 (0.3)^§^
	*ZEB-2*, gene expression	2.3 (1.3)	3.5 (2.7)	0.7 (0.2)^#^	0.4 (0.3)^§§§^
miR-126					
	*EGFL-7*, gene expression	4.0 (2.0)	5.0 (3.0)	5.0 (5.0)	1 (0.7)
	*TOM-1*, gene expression	0.6 (0.3)	2.2 (1.1)^***^	1.3 (1.0)	0.8 (0.6)^§§§^
	*CRK*, gene expression	8.6 (7.1)	21.3 (19.7)^*^	6.6 (5.8)	3.7 (3.5)^§§§^
	Ang-2, protein content	0.3 (0.1)	0.3 (0.2)	0.3 (0.07)	0.5 (0.2)^*, §^
	Fibulin-3, protein content	0.6 (0.2)	0.6 (0.3)	0.5 (0.2)	0.4 (0.2)
	Fibulin-2, protein content	0.04 (0.03)	0.1 (0.04)^*^	0.04 (0.02)	0.04 (0.03)
	Fibulin-5, protein content	0.3 (0.1)	0.4 (0.1)	0.5 (0.1)^#^	0.5 (0.1)
miR-451					
	*MIF*, gene expression	0.6 (0.8)	2.5 (2.1) ^**^	1.3 (1.1)	0.3 (0.2) ^§§§^
	*RAB-14*, gene expression	6.3 (5.1)	4.1 (1.9)	1.5 (1.3) ^##^	2.4 (2.1)
miR-210					
	*FGFRL-1*, gene expression	2.5 (1.8)	3.2 (2.0)	4.1 (2.3)	1.3 (1.0)^***, §^
	*EFNA-3*, gene expression	0.3 (0.2)	1.0 (0.9)^*^	0.2 (0.1)	0.1 (0.08)^§§^
	P-62, protein content	0.1 (0.04)	0.3 (0.1)^*^	0.1 (0.02)	0.3 (0.1)^*^
	LC3II/LC3II, protein content	0.1 (0.05)	0.4 (0.1)^***^	0.2 (0.1)	0.4 (0.1)^*^
	Beclin-1, protein content	0.3 (0.09)	0.3 (0.05)	0.2 (0.1)	0.3 (0.1)
	BAX, protein content	0.2 (0.06)	0.3 (0.2)	0.2 (0.1)	0.3 (0.2)
	BCL-2, protein content	0.04 (0.02)	0.2 (0.1)^***^	0.1 (0.04)	0.2 (0.05)^**^
miR-30a-30p					
	*SNAIL-1*, gene expression	2.3 (1.4)	3.4 (2.8)	2.8 (1.7)	0.6 (0.4)^*, §§§^
	*P53*, gene expression	0.4 (0.3)	0.7 (0.5)	0.5 (0.3)	0.3 (0.2)^§^
	*CDKN2A*, gene expression	4.7 (3.9)	1.9 (1.8)^*^	9.8 (7.8)^#^	19.4 (16.5)^§§§^
	*P63*, gene expression	0.1 (0.08)	0.1 (0.05)	0.8 (0.5)^###^	0.4 (0.3)^*^
	*CDKN1A*, gene expression	11.8 (10.4)	5.7 (3.5)^*^	13.5 (7.7)	6.4 (4.8)^**^
	Ki-67 positive cells, %	42.6 (19.0)	96.5 (3.0)^***^	42.6 (19.0)	96.2 (4.7)^***^
miR-let7c					
	*k-RAS*, gene expression	3.0 (2.0)	2.2 (1.0)	2.7 (2.1)	1.1 (0.5)^*, §^

Variables are represented as mean (standard deviation)

*Abbreviations*: *miR* microRNA, *rel.expresion* relative expression, *prot.content* protein content, *PTEN* phosphatase and tensin homolog 10, *MARCKS* myristoylated alanine-rich protein kinase C substrate, *TPM1* tropomyosin 1 (alpha), *PDCD4* programmed cell death 4, *SPRY2* sprouty homolog 2, *ETS1* v-ets avian erythroblastosis virus E26 oncogene homolog 1, *ZEB2* zinc finger E-box binding homeobox 2, *EGFL7* EGF-like-domain, multiple 7, *TOM1* target of myb1, *CRK* v-crk avian sarcoma virus CT10 oncogene homolog 17, *Ang-2* angiopoietin-2, *MIF* macrophage migration inhibitory factor, *RAB14* member RAS oncogene family 14, *FGFRL-1* fibroblast growth factor receptor-like 1, *EFNA3* ephrin-A3, *LC3* light chain 3, *BAX* bcl-2 associated X protein, *BCL-2* B cell lymphoma-2, *SNAIL-1* snail family zinc finger 1, *P53* tumor protein p53, *CDKN2A* cyclin-dependent kinase inhibitor 2A, *P63* protein p63, *CDKN1A* cyclin-dependent kinase inhibitor 1A, and *k-RAS* Kirsten rat sarcoma viral oncogene homolog 11

^§^*p* < 0.05, ^§§^*p* < 0.01, ^§§§^*p* < 0.001 between tumor lungs of LC-COPD and tumor specimens of LC patients; ^*^p < 0.05, ^**^p < 0.01, ^***^p < 0.001 between tumor and non-tumor lungs in either LC or LC-COPD patients; ^#^*p* < 0.05, ^##^*p* < 0.01, ^###^*p* < 0.001 between non-tumor specimens of LC-COPD and non-tumor lungs of LC patients. Absence of any statistical symbol means no differences between the study groups

**Table 3:** Summary of epigenetic events in the tumor specimens of LC patients with and without the chronic respiratory condition

Epigenetic events	LC patients	
	Non-COPD	COPD
Lung tumors		
MicroRNAs		
miR-21, miR-200b, miR-210, miR-let7c	**↓**	↑
miR-126, miR451, miR30a-30p, miR-155, miR-let7a	**=**	=
Histone modifications		
HDAC2, *Sirtuin-1*	**=**	=
DNA methylation	**↓**	↑

*Abbreviations*: *miR* microRNA, *HDAC2* histone deacetylase-2, = there were no significant differences for the specific tumor markers between LC-COPD and LC patients without COPD

**Table 4:** Summary of biological processes represented by the downstream markers of the epigenetic events explored in the study in the tumor specimens of LC patients with and without COPD

Downstream markers	LC patients	
	Non-COPD	COPD
Tumor suppressor genes *P53*, *PTEN*, *PDCD4*, *TPM-1*, *k-RAS* *CDKN2A* *Sirtuin-1*	↑	**↓**
	**↓**	↑
	**=**	=
Cell proliferation and invasion (through ↓ *SPRY-2*, *MIF* and *TOM-1*)	**↓**	↑
Apoptosis (through ↓ *ETS-1*)	↑	**↓**
Cell differentiation (through ↓ *ETS-1* and *FGFRL-1*)	↑	↓
Epithelial mesenchymal transition (through ↓ *ZEB2* and *SNAIL-1*)	↑	↓
Cell- mediated immunity (through ↓ *MIF*)	↓	↑
Angiogenesis (through ↑ angiopoietin-2 and ↓ EFNA-3)	↓	↑
Cell adhesion and migration (through ↓ *CRK*, *TOM-1* and *MARCKs*)	↑	↓

= there were no significant differences for the specific tumor markers between LC-COPD and LC patients without COPD

#### Methylated DNA

Global 5-methylcytosine was quantified using the MethylFlash Methylated Kit and previous studies [30, 34].

#### Immunoblotting of 1D electrophoresis

Protein levels of the different molecular markers in lung specimens were explored using methodologies previously published [11, 31–33, 35].

#### Immunohistochemistry

Levels of ki-67 (marker of cell proliferation) were identified in the lung specimens as previously described [11, 31, 32, 35].

### Statistical analyses

Data are expressed as mean (standard deviation). Normality of the variables was explored using the Shapiro-Wilk test. For the quantitative variables in the lungs, differences between study groups were assessed using one-way analysis of variance (ANOVA) and Tukey’s post hoc analysis to adjust for multiple comparisons of all the study variables. Chi-square test was used to assess potential differences between the study groups for the qualitative variables. Statistical significance was established at *P* ≤ 0.05.

## Results

### Clinical characteristics

Clinical and functional characteristics of LC-COPD and LC patients recruited in the study are shown in Table 1. Age, gender, and BMI did not significantly differ between LC-COPD and LC patients (Table 1). Interestingly, smoking history was similar in both study groups (Table 1). The functional parameters forced expiratory volume in 1 s (FEV_1_), FEV_1_/forced vital capacity (FVC), and diffusion lung capacity for carbon monoxide (DL_CO_) were significantly lower in LC-COPD than LC patients (Table 1). Furthermore, no significant differences were found in either TNM or histological subtypes between LC-COPD and LC groups (Table 1). In LC-COPD compared to LC patients, levels of albumin and fibrinogen were significantly decreased, whereas those of globular sedimentation (GSV) were increased and C-reactive protein (CRP) levels did not differ between groups (Table 1).

### Differential microRNA profile and downstream targets in lung tumors in patients with underlying respiratory disease

**Fig. 1:**
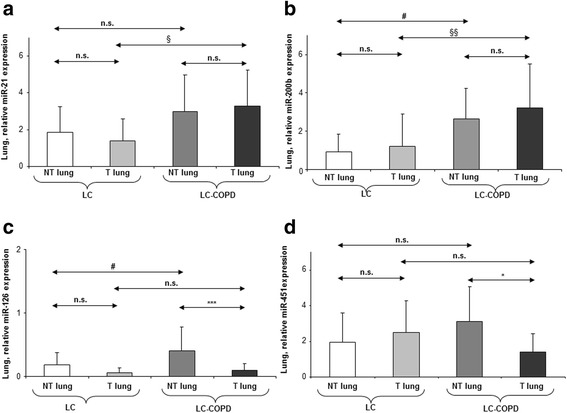
Mean values and standard deviation (relative expression) of miR-21 (**a**), miR-200b (**b**), miR-126 (**c**), and miR-451 (**d**). Definition of abbreviations: miR, microRNA. Statistical significance: *p < 0.05, ***p < 0.001 between tumor and non-tumor lungs in either LC or LC-COPD patients; ^#^*p* < 0.05 between non-tumor specimens of LC-COPD and non-tumor lungs of LC patients; ^§^*p* < 0.05, ^§§^*p* < 0.01 between tumor lungs of LC-COPD and tumor specimens of LC patients; and n.s., non-significant differences between the study groups

In LC-COPD compared to LC patients, miR-21 expression was significantly increased in the tumors, whereas that of its downstream targets *PTEN*, *MARCKs*, *TPM-1*, *PDCD4*, and *SPRY-2* was significantly reduced (Fig. 1a and Table 2). However, non-tumor lung expression of miR-21, *PTEN*, *MARCKs*, *TPM-1*, *PDCD4*, and *SPRY-2* did not significantly differ between LC-COPD and LC patients (Fig. 1a and Table 2). Tumor miR-200b expression was significantly greater in LC-COPD than in LC patients, while that of its downstream markers *ETS-1* and *ZEB-2* was significantly reduced (Fig. 1b and Table 2). Non-tumor lung expression of miR-200b also significantly increased in LC-COPD compared to LC patients, and only the expression of *ZEB-2* significantly decreased (Fig. 1b and Table 2). Tumor expression levels of miR-126 did not significantly differ between LC-COPD and LC patients, whereas in the former patients, an almost significant decrease in *EGFL-7* expression was observed (*p* = 0.073, Fig. 1c and Table 2), together with a decrease in *TOM-1* and *CRK* expression and a rise in angiopoietin-2 content (Table 2). Tumor protein levels of fibulin-3, fibulin-2, and fibulin-5 did not differ between LC-COPD and LC patients (Table 2 and Additional file 1: Figure S1).

**Fig. 2:**
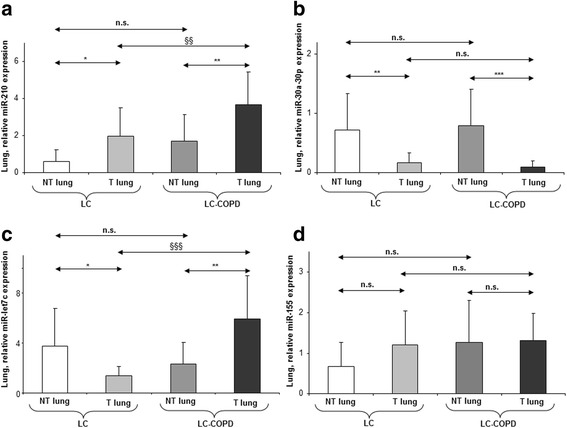
Mean values and standard deviation (relative expression) of miR-210 (**a**), miR-30a-30ap (**b**), miR-let7c (**c**), miR-155 (**d**), and miR-let7a (**e**) in tumor (T) and non-tumor (NT) lungs of LC and LC-COPD patients. Definition of abbreviations*:* miR, microRNA. Statistical significance: *p < 0.05, **p < 0.01, ***p < 0.001 between tumor and non-tumor lungs in either LC or LC-COPD patients; ^§§^*p* < 0.01, ^§§§^*p* < 0.001 between tumor lungs of LC-COPD and tumor specimens of LC patients; and n.s., non-significant differences between the study groups

**Fig. 3:**
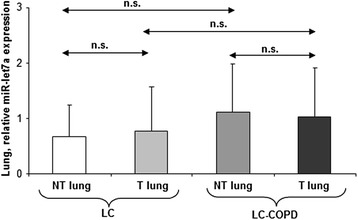
Mean values and standard deviation (relative expression) of miR-let7a in tumor (T) and non-tumor (NT) lungs of LC and LC-COPD patients. Definition of abbreviations*:* miR, microRNA. Statistical significance: n.s. non-significant differences between the study groups

Tumor and non-tumor expression of miR-451 did not differ between LC-COPD and LC patients (Fig. 1d), while *MIF* tumor expression was significantly lower in LC-COPD than LC patients, and RAB-14 expression was also significantly reduced in the non-tumors of the former patients (Table 2). Tumor expression of miR-210 significantly increased in LC-COPD compared to LC patients, while that of its downstream targets *FGFRL-1* and *EFNA-3* was reduced in the former patients (Fig. 2a and Table 2). Tumor protein levels of autophagy and apoptosis markers P62, LC3II/LC3I, beclin-1, BAX, and BCL-2 did not differ between LC-COPD and LC patients (Table 2 and Additional file 1: Figure S2). In LC-COPD, tumor expression of miR30a-30p was similar to that seen in LC patients (Fig. 2b). However, tumor expression of its downstream markers *SNAIL-*1 and *P53* was significantly lower in LC-COPD than LC patients, while that of *CDKN2A* was greater and that of *P63*, *CDKN1A*, and ki-67 did not differ in tumors between patient groups (Table 2 and Additional file 1: Figure S3). Furthermore, non-tumor lung expression levels of miR-210 and miR30a-30p and their downstream markers *FGFRL-1*, *EFNA-3*, P62, LC3II/LC3I, beclin-1, BAX, BCL-2, *SNAIL-1*, *P53*, *CDKN1A*, and ki-67 did not significantly differ between LC-COPD and LC patients (Fig. 2a, b, Additional file 1: Figures S2, S3 and Table 2). Nonetheless, non-tumor lung expression of *CDKN2A* and *P63* was significantly higher in LC-COPD than LC patients (Table 2). Tumor expression of miR-let7c was significantly greater in LC-COPD than LC patients (Fig. 2c), while that of its downstream marker *k-RAS* significantly decreased in the former patients (Table 2). Tumor expression of miR-155 and miR-let7a did not significantly differ between LC-COPD and LC patients (Figs. 2d and 3, respectively). Additionally, non-tumor lung expression levels of miR-let7c, *k-RAS*, miR-155, and miR-let7a markers did not significantly differ between LC-COPD and LC patients (Figs 2c, d and 3 and Table 2).

### Differential profile of histone deacetylases and DNA methylation in lung tumors of patients with underlying chronic respiratory disease

**Fig. 4:**
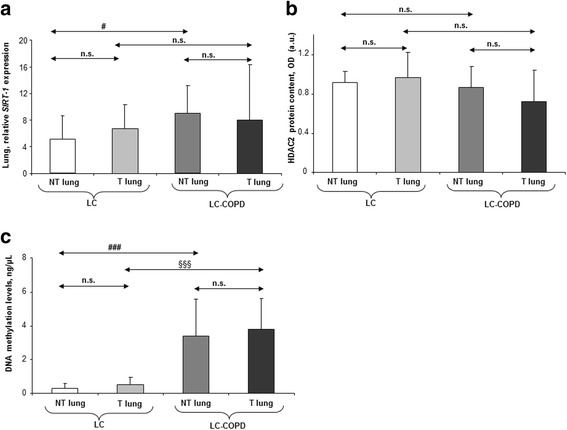
Mean values and standard deviation (relative expression) of *SIRT-1* (**a**), HDAC2 (**b**), and DNA methylation levels (**c**) in tumor (T) and non-tumor (NT) lungs of LC and LC-COPD patients. Definition of abbreviations: miR, microRNA; *SIRT-1*, sirtuin-1; and HDAC2, histone deacetylase 2. Statistical significance: ^#^*p* < 0.05, ^###^*p* < 0.001 between non-tumor specimens of LC-COPD and non-tumor lungs of LC patients; ^§§§^*p* < 0.001 between tumor lungs of LC-COPD and tumor specimens of LC patients; and n.s., non-significant differences between the study groups

Tumor expression levels of the markers *SIRT-1* and histone deacetylase-2 (HDAC2) did not differ between the two study groups (Fig. 3a, b and Additional file 1: Figure S4). Nevertheless, in LC-COPD compared to LC patients, non-tumor *SIRT-1* expression was greater, whereas HDAC2 non-tumor protein levels did not differ between the study groups (Fig. 4a, b and Additional file 1: Figure S4). Total DNA methylation levels were significantly greater in both tumor and non-tumor lungs of LC-COPD than in LC patients (Fig. 4c).

## Discussion

In the current study, the study hypothesis has been confirmed. Importantly, the most relevant findings encountered in the investigation were that expression levels of miR-21, miR-200b, miR-210, miR-let7c, and DNA methylation were significantly greater in lung tumors of patients with underlying COPD than in those without this chronic condition (Table 3). Furthermore, expression levels of the downstream markers of the corresponding microRNAs, namely *PTEN*, *MARCKs*, *TPM-1*, *PDCD4*, *SPRY-2*, *ETS-1*, *ZEB-2*, *FGFRL-1*, *EFNA-3*, and *k-RAS* were downregulated in the lung tumor lesions of patients with the underlying condition compared to non-COPD patients (Table 4). Importantly, levels of the tumor suppressor gene *P53* were also downregulated in the tumors of patients with COPD. These are biological markers of the underlying processes that are involved in tumorigenesis and tumor progression. Remarkably, these are novel findings that may partly explain the underlying biology of the reported greater risk of patients with COPD to develop lung tumors.

Downregulation of the study genes may entail alterations in the target pathways such as increased cell proliferation, migration, adhesion, invasion and angiogenesis, potential development of metastatic tumors, and adaptation of the cancer cells to hypoxia [25]. Clearly, all these biological processes promote tumor growth and favor disease progression in the patients. Previous investigations have demonstrated that levels of miR-21, miR-200b, miR-210, and miR-let7c were also upregulated in the tumor lesions of lung cancer cells in several experimental models and in patients [25, 36–42]. Interestingly, in other investigations, cancer cells were also shown to be more resistant to radiation or chemotherapy when those markers were elevated [43, 44]. In the present investigation, these results suggest that upregulation of miR-21, miR-200b, miR-210, and miR-let7c expression and that of DNA methylation may partly underlie the increased susceptibility of COPD patients to develop lung tumors. In keeping with, aberrant methylation status of tumor suppressor (*CDKN2A*) and DNA repair genes has already been identified in tumors of patients with LC and COPD [45, 46]. Furthermore, it has also been proposed that cancer cells express specific patterns of microRNA profiles that may contribute to the process of tumorigenesis [46]. Whether these epigenetic events may also confer a greater resistance to cancer treatment in patients with COPD will remain a matter of future research.

As the approach used in the study enabled us to analyze the target markers also in the surrounding non-tumor parenchyma in both groups of patients with LC, it is worth mentioning that levels of expression of miR-200b, miR-126, and DNA methylation were significantly greater in non-tumor specimens of patients with COPD than in those without this chronic condition. Additionally, expression levels of the downstream markers *ZEB-2* and *RAB-14* were downregulated, while those of the tumor suppressors *CDKN2A*, *P63*, and *SIRT-1* were upregulated in the non-tumor specimens of LC patients with underlying respiratory disease compared to non-COPD patients. These are relevant findings that reveal the underlying biology of the lung parenchyma in patients with COPD. These results also imply that in COPD, a differential epigenetic regulation of cell cycle and proliferation also takes place in the lung parenchyma regardless of the presence of cancer cells. In fact, it has been clearly demonstrated that transition of cancer cells at the invasive site to a mesenchyme-like phenotype, commonly known as epithelial-to-mesenchymal transition, is crucial for invasion and distant metastasis in cancer progression [47]. In the current investigation, expression levels of the epithelial-to-mesenchymal transition inducers *SNAIL*-1 and *ZEB-*2 were significantly downregulated in tumors of patients with LC-COPD compared to those without underlying COPD. Thus, these results may imply that epithelial-to-mesenchymal transition may not play a major role in lung tumorigenesis in those patients, probably as result of areas of lung destruction. Clearly, future avenues of research should specifically address this question.

A third group of novel findings observed in this investigation were that within each study group of patients, in the lung tumors compared to non-tumor specimens, expression levels of miR-126 and miR-451 decreased in LC-COPD patients, while those of miR-30a-30p were reduced in both groups of patients. Compared to non-tumor specimens, levels of miR-let7c were also lower in lung tumors of patients without COPD, while a rise was seen in those with underlying COPD. The last findings are somehow counter to those previously reported in patients with COPD, who exhibited a decrease in miR-let7c expression in the sputum macrophages and bronchial airway epithelial cells [24]. As miR-let7c acts a tumor suppressor in tumor cells, it is likely that COPD induces a positive feedback on its expression in order to offset the deleterious effects of cancer biology. Levels of miR-let7c, which negatively correlated with metastasis, vascular invasion, and poor survival, were also downregulated in patients with NSCLC in another investigation [42]. However, whether patients also had underlying chronic respiratory disease was not explored in that study [42].

Posttranslational modifications of histones regulate gene transcription in normal and disease-related cellular processes. Levels of markers regulated by histone acetylation such as *PTEN*, *MARCKs*, EGFL-7 (almost significant decrease), *FGFRL-1*, *SNAIL-1*, *P63*, and *k-RAS* were significantly reduced in the lung tumors compared to non-tumors only in patients with LC-COPD. Indeed, proliferation markers and *k-RAS* mutations were reduced in lung tumors among COPD patients [48]. However, expression levels of downstream markers *TPM-1*, *TOM-1*, *CRK*, fibulin-2, *MIF*, and *EFNA-3* were greater in the lung tumors than in non-tumor specimens only in patients without COPD. Interestingly, in the tumors of both groups of patients, the autophagy markers P62 and LC3, the anti-apoptotic BCL-2, and the proliferation marker ki-67 were increased compared to non-tumor specimens, while those of the inhibitor of cell proliferation *CDKN1A* were reduced. These findings also confirm that epigenetics play a significant role in the regulation of cellular mechanisms such as apoptosis, angiogenesis, and cell migration and adhesion that underlie tumorigenesis itself in both groups of patients, with a specific pattern of expression in patients with COPD.

### Study critique

Since epigenetic events may be influenced by cigarette smoking, results might differ between smokers and non-smokers. Nonetheless, as no differences in smoking history or pack-years were observed between the two study groups, the results encountered in the investigation are likely to be attributed to the presence of the lung tumor and COPD in the patients with the underlying condition. Another factor that might have affected the study results is aging. Nevertheless, as age did not significantly differ between the two patient groups, the encountered findings can also be mostly recognized to the presence of the neoplasm and to COPD in the patients with this disease. Another limitation in the study refers to the relatively small number of lung specimens analyzed from both patient groups. Nevertheless, for ethical reasons, tumor and non-tumor lung specimens could only be obtained from patients undergoing thoracotomy for the treatment of their lung neoplasm (approximately 25% of all patients with LC). It should also be acknowledged that in three patients of the LC group, the lung neoplasms were in stage IV. These patients underwent surgery, chemotherapy, and/or radiotherapy for the treatment of the single metastasis always following thoracotomy for their primary lung neoplasm. It should also be mentioned that the approach used in the investigation enabled us to assess the effects of COPD itself without the cancer lesions on the epigenetic targets as distant non-tumor samples were also obtained from both groups of study patients. This is a unique approach commonly used in our group and the only acceptable from an ethical standpoint as also previously reported [11, 14]. Finally, future studies may be conducted in which the markers analyzed in this study will be validated in large-scale cohorts of patients. Nonetheless, it should also be emphasized that the results reported in the present investigation have been obtained within the frame of a hypothesis-driven prospective study.

### Clinical impact of the study findings and future perspectives

Epigenetic regulation of cellular processes may explain disease pathogenesis. In the study, LC patients with an underlying respiratory condition exhibited a differential expression profile of several epigenetic markers, namely DNA methylation and microRNA expression along with that of downstream markers that are involved in tumorigenesis and tumor suppression. The clinical relevance of these findings is based on the following principles: (1) the target biological processes analyzed in the study may be eventually used as potential biomarker panels for the early diagnosis of LC in patients with COPD (the vast majority of patients with LC); (2) the epigenetic mechanisms could be used as potential biomarkers of disease progression (clinical staging) and prognosis, especially in patients with chronic respiratory diseases; (3) microRNAs such as miR-21 and miR-let7c may be targeted pharmacologically [49, 50], and thus, they may act as suitable biomarkers for the treatment of LC, which in turn may also help to gauge the response to therapy in these patients; and (4) the presence of an underlying respiratory disease should be identified in all patients with LC as the differential biological profile may help determine tumor progression and the therapeutic response. Future investigations should focus on the elucidation of whether microRNAs may serve as biomarkers of tumor progression, clinical staging, and recurrence in patients with underlying respiratory diseases.

## Conclusions

A differential epigenetic regulation characterized by increased expression of several microRNAs and DNA methylation that control cellular processes involved in lung tumorigenesis exists in patients with LC and underlying respiratory disease. Fingerprint biomarkers of mechanisms involved in tumor growth, angiogenesis, migration, and apoptosis were also differentially expressed in tumors of patients with COPD. These findings shed light into the underlying biology of the reported greater risk to develop LC in patients with chronic respiratory conditions. Furthermore, they also have potential therapeutic implications as epigenetic events may be specifically targeted pharmacologically.

## Additional file

Additional file 1: Detailed information on the methodologies used in the study as well as the immunoblot images of the analysed markers. (DOCX 1563 kb)

## Abbreviations

ANG-2: Angiopoietin-2
ANOVA: One-way analysis of variance
BAX: Bcl-2-associated X protein
BCL-2: B cell lymphoma-2
BMI: Body mass index
*CDKN1A*: Cyclin-dependent kinase inhibitor 1A
*CDKN2A*: Cyclin-dependent kinase inhibitor 2A
COPD: Chronic obstructive pulmonary disease
*CRK*: v-crk avian sarcoma virus CT10 oncogene homolog 17
CRP: C-reactive protein
DLco: Carbon monoxide transfer
*EFNA3*: Ephrin-A3
*EGFL7*: EGF-like-domain, multiple 7
*ETS1*: v-ets avian erythroblastosis virus E26 oncogene homolog 1
FEV_1_: Forced expiratory volume in the first second
*FGFRL-1*: Fibroblast growth factor receptor-like 1
FVC: Forced vital capacity
GAPDH: Glyceraldehyde-3-phosphate dehydrogenase
GSV: Globular sedimentation velocity
HDAC2: Histone deacetylase-2
K_CO_: Krogh transfer factor
*k-RAS*: Kirsten rat sarcoma viral oncogene homolog 11
L: Liter
LC: Lung cancer
LC3: Light chain 3
*MARCKS*: Myristoylated alanine-rich protein kinase C substrate
*MIF*: Macrophage migration inhibitory factor
miR: MicroRNA
*N*: Number
NSCLC: Non-small cell lung cancer
*P53*: Tumor protein p53
*P63*: Protein p63
*PDCD4*: Programmed cell death 4
*PTEN*: Phosphatase and tensin homolog 10
qRT-PCR: Quantitative real time polymerase chain reaction
*RAB14*: Member RAS oncogene family 14
SD: Standard deviation
*SIRT-1*: Sirtuin-1
*SNAIL-1*: Snail family zinc finger 1
*SPRY2*: Sprouty homolog 2
TNM: Tumor, nodes, metastasis
*TOM1*: Target of myb1
*TPM1*: Tropomyosin 1 (alpha)
*ZEB2*: Zinc finger E-box binding homeobox 2
