# Supplementary material for: Profile of epigenetic mechanisms in lung tumors of patients with underlying chronic respiratory conditions
Source: Clin Epigenetics. 2018 Jan 16;10:7. doi: 10.1186/s13148-017-0437-0 (PMC5771157; doi:10.1186/s13148-017-0437-0)

**ONLINE SUPPLEMENTARY MATERIAL**

**profile of epigenetic mechanisms in lung TUMORS of patients with underlying CHRONIC RESPIRATORY DISEASE**

**Mercè Mateu-Jimenez, Víctor Curull, Alberto Rodríguez-Fuster, Rafael Aguiló, Albert Sánchez-Font, Lara Pijuan, Joaquim Gea****, Esther Barreiro**

**METHODS**

**Study patients**

This was a prospective, controlled study in which a group of 40 Caucasian patients (33 males, Table 1) with LC, undergoing thoracotomy for their lung neoplasm, were recruited consecutively from the Lung Cancer Clinic of the Respiratory Medicine Department at *Hospital del Mar* (Barcelona, Spain). Patients were always recruited before having received any treatment for their lung neoplasm including chemotherapy and/or radiotherapy. Tumor and non-tumor lung specimens were obtained from all patients and were subdivided into two groups depending on the presence of underlying COPD or not, which was diagnosed on the basis of current guidelines (1-3): 1) 20 patients with LC who also had COPD (LC-COPD group) and 2) 20 patients with LC without COPD (LC group). As part of the prospective ongoing cohort “*Lung Cancer-COPD*” in our center, both LC and LC-COPD patients simultaneously participated in a previous study aimed to assess Th1 and Th2 inflammatory profiles and their implications in lung tumors of patients with chronic respiratory conditions(4).

Histological diagnosis and staging, (tumor, node, metastasis, TNM) of LC were confirmed in all patients. At study entry, exclusion criteria were as follows: previous chemotherapy or radiotherapy, chronic cardiovascular, metabolic, and clot system disorders, signs of severe bronchial inflammation and/or infection (bronchoscopy), current or recent invasive mechanical ventilation, and long-term oxygen therapy. In the current investigation, approval was obtained from the institutional Ethics Committee on Human Investigation (*Hospital del Mar*–*IMIM*, Barcelona) in accordance with the World Medical Association guidelines (Helsinki Declaration of 2008) for research on human beings. Informed written consent was obtained from all patients at study entry and prior to initiation of therapies.

**Anthropometrical and Functional Assessment**

Lung function was evaluated in all patients through determination of spirometric values, static lung volumes, diffusion capacity, and blood gases using standard procedures. Body composition was evaluated by the assessment of body mass index (BMI). Moreover, nutritional parameters were also evaluated through conventional blood tests (4-9).

### Sample collection

During the surgical intervention (minimal resection or lobectomy), in patients that underwent thoracotomy (n=40), lung samples were obtained (tumor and non-tumor) specimens by the expert lung pathologist, from the distant surrounding parenchyma (7 cm minimum distance from the tumor site in all the samples) with respect to where the tumor was localized, which enabled us to study non-tumor lung specimens. Briefly, non-tumor samples were carefully selected as a result of visual and manual detection (palpation) of any potential nodules that might modify disease staging. The presence of ground-glass opacities in the non-tumor specimens was always ruled out by previously analyzing the CT scan sections in all the study patients. Additionally, hematoxylin-eosin staining was performed in all non-tumor lung specimens to further confirm the absence of cancer cells. For ethical reasons this was the only approach approved to study non-tumor lungs in COPD and non-COPD patients in our institution. In all cases, the expert lung pathologist selected a fragment of lung tumor and non-tumor specimens of approximately 10x10 mm^2^ size from the fresh samples after a careful collection of the specimens required for diagnosis purposes. On the day of thoracotomy, blood samples were obtained for conventional routine blood tests (hemogram and nutritional parameters). Importantly, a minimum amount of 50% of cancer cells was similarly identified in all tumor types from all the study patients. The remaining cell components were inflammatory and stromal cells in all the analyzed tumors. On the day of thoracotomy, blood samples were obtained for conventional routine blood tests (hemogram and nutritional parameters).

### Sample preservation

Lung specimens (tumor and non-tumor) were snap-frozen in liquid nitrogen and stored at −80 °C for further use.

### Molecular biology analyses

### The biology analyses were all conducted blind in our molecular biology laboratory (IMIM).

*RNA isolation***.** Total RNA was isolated from snap-frozen lung specimens (tumor and non-tumor) using the Trizol reagent and following the manufacturer’s protocol (Life technologies, Carlsbad, CA, USA). Total RNA concentrations were determined spectrophotometrically using the NanoDrop 1000 (Thermo Scientific, Waltham, MA, USA).

*MicroRNA reversed transcription (RT).* MicroRNA RT was performed using TaqMan microRNA assays (Life Technologies) following the manufacturer’s instructions. First-strand cDNA was generated from mRNA using oligo(dT)_12-18_ primers and the Super-Script^TM^ III reverse transcriptase following the manufacturer’s instructions (Life technologies).

*Quantitative real time-PCR amplification (qRT-PCR)*. TaqMan based qPCR reactions were performed using the ABI PRISM 7900HT Sequence Detector System (Applied BioSystems, Foster City, CA, USA) together with commercially available predesigned microRNA assays, primers, and probes for the genes corresponding to specific downstream markers (involved in key cellular processes related to tumorigenesis and cancer progression, Tables E1, E2, 2 and 3) of the microRNAs analyzed in the current study: *PTEN*, *PDCD4*, *TPM1*, *SPRY-2*, *MARCKS*, *ETS1*, *ZEB-2*, *EGFL7*, *CRK*, *TOM-1*, *MIF*, *RAB-14*, *FGFRL1*, *EFNA3*, *SNAIL1*, *CDKN1A, CDKN2A*, *P53*, *P63*, and *k-RAS*. Taqman microRNA assay for small nuclear RNA U6 (snU6) was used in order to normalize the miRNA amplifications and the housekeeping gene glyceraldehyde-3-phosphate dehydrogenase (GAPDH) was used as the endogenous control for mRNA gene expression. MicroRNA and mRNA data were collected and analyzed using the SDS Relative Quantification Software version 2.1 (Applied BioSystems), in which the comparative C_T_ method (2-ΔΔCT) for relative quantification was employed (10). Samples were always run in triplicates and their corresponding expression was calculated as the mean value of the 3 measurements (7;9;11;12).

*DNA isolation***.** Total DNA was isolated from lung specimens (tumor and non-tumor) of all study patients using QIAmp DNA Mini Kit (QiAgen, Redwood City, CA, USA) (9;11;12), following the manufacturer’s protocol of DNA purification from tissues, and without the use of ribonuclease-A. Total DNA obtained from the lung specimens (tumor and non-tumor) was quantified using a spectrophotometer (NanoDrop, Thermo Scientific, Wilmington, DE, USA).

*Quantification of methylated DNA using enzyme-linked immunosorbent assay (ELISA)-based immunoassay.* Global 5-methylcytosine (5-mC) in DNA was quantified in lung specimens (tumor and non-tumor) of all the study patients (LC and LC-COPD) using the MethylFlash Methylated DNA Quantification Colorimetric Kit (Epigentek, Farmingdale, NY, USA) following the manufacturer’s instructions and previous studies (7;12). Briefly, 80 μL of binding solution were first added into each plate well. Then, 100 ng of each DNA sample and positive and negative controls were added in duplicates in the corresponding plate wells and covered with an adhesive tape. The plate was incubated at 37º for one hour and a half, washed and then incubated again with 50 μL of the specific antibody for one hour at room temperature. Then, after several washes, 50 μL of a specific detection antibody was added and the plates were incubated at room temperature for 30 minutes. Samples were then washed several times, and 50 μL of enhanced solution were added to the plates for another 30-minute incubation at room temperature. Finally, after several washes, 100 μL of developing solution were added, and the enzyme reaction was stopped using a specific stop solution. Absorbances were read at 450 nm in a microplate spectrophotometer (Microplate reader M680, Bio-Rad, Hercules, CA, USA) using a reference filter of 655 nm. A standard curve was generated with each assay run and sample concentrations were calculated based on the manufacturer’s instructions. The minimum detectable concentration in the samples was set to be 0.2 ng of methylated DNA (Epigentek). The final methylated DNA concentration was expressed as the mean value of the two measurements from the duplicates. Data are expressed as the percentage of total methylated DNA to total DNA in the lung samples (both tumor and non-tumor specimens).

*Immunoblotting of 1D electrophoresis.* Protein levels of the different molecular markers in lung specimens were explored using methodologies previously published (6;8;9;11;13). Briefly, frozen lung samples from the study patients were homogenized in a buffer containing 50 mM HEPES, 150 mM NaCl, 100 mM NaF, 10 mM Na pyrophosphate, 5 mM EDTA, 0.5% Triton-X, 2 μg/ml leupeptin, 100 μg/ml PMSF, 2 μg/ml aprotinin and 10 μg/ml pepstatin A. Samples were then centrifuged at 1,000 G for 30 minutes. The supernatant was designated as the crude cytoplasmic homogenate and the pellet was discarded. The entire procedures were always conducted at 4ºC (on ice). Protein levels in each homogenate were spectrophotometrically determined with the Bradford procedures using triplicates in each case and bovine serum albumin (BSA) as the standard (Bio-Rad protein reagent, Bio-Rad Inc., Hercules, CA, USA). The final protein concentration in each sample was calculated from at least two Bradford measurements that were almost identical. Equal amounts of total protein from crude lung homogenates (ranging from 10 to 30 μg per sample/lane) were always loaded onto the gels, as well as identical sample volumes/lanes. For the sake of the comparisons among the study groups, sample specimens were always run together and kept in the same order.

SDS-PAGE gels were used to detect protein levels of different markers in the lung specimens (tumor and non-tumor) from both groups of patients (LC and LC-COPD). The gels were always run in the same electrophoresis box for each of the antigens. Fresh gels containing the study samples were specifically loaded for each of the antigens. Proteins were then separated by electrophoresis, transferred to polyvinylidene difluoride (PVDF) membranes, blocked with non-fat milk 5% or with bovine serum albumin 1% and incubated with selective primary antibodies overnight. The following antibodies were used in order to detect the different molecular markers: histone deacetylase 2 (HDAC2, anti-HDAC2 antibody, Santa Cruz Biotechnology, Santa Cruz, CA, USA), P62 (anti-P62/SQSTM1 antibody, Sigma-Aldrich), beclin-1 (anti-beclin-1 antibody, Santa Cruz), light-chain 3 (LC3B, anti-LC3B antibody, Cell Signaling), bcl-2 associated X protein (BAX) (BAX, anti-BAX antibody, Santa Cruz), b-cell lymphoma 2 (BCL-2, anti-BCL-2, antibody Santa Cruz), fibulin-3 (anti-fibulin-3 antibody, Santa Cruz), fibulin-5 (anti-fibulin-5 antibody, Santa Cruz), fibulin-2 (anti-fibulin-2 antibody, Santa Cruz), angiopoietin-2 (anti-angiopoietin-2 antibody, Santa Cruz) and glyceraldehyde-3-phosphate dehydrogenase (GAPDH, anti-GAPDH antibody, Santa Cruz). Antigens from all samples were detected with HRP-conjugated secondary antibodies and a chemiluminescence kit. The specificity of the different antibodies was confirmed by omission of the primary antibody, and incubation of the membranes only with secondary antibodies.

PVDF membranes were scanned with the Molecular Imager Chemidoc XRS System (Bio–Rad Laboratories, Hercules, CA, USA) using the software Quantity One version 4.6.5 (Bio–Rad Laboratories). Optical densities of specific proteins were quantified using the software Image Lab version 2.0.1 (Bio-Rad Laboratories). Final optical densities obtained in each specific group of patients corresponded to the mean values of the different samples (lanes) of the study antigens. To validate equal protein loading among various lanes, SDS-PAGE gels were stained with Coomassie Blue and the glycolytic enzyme GAPDH was used as the protein loading controls in all the immunoblots.

*Immunohistochemistry.* In the tumor and non-tumor lung specimens (N=8/group) of each group (LC and LC-COPD), the proliferation marker ki-67 (anti-ki67 antibody, Millipore Iberica, CA, USA) was identified on the three-micrometer tumor and non-tumor paraffin-embedded sections using immunohistochemical procedures as previously described in our group (6;8;9;13). Briefly, lung specimen cross-sections were deparaffinized and antigen retrieval was carried after heating slides in a water bath in EDTA buffer pH 8. Slides were then treated with 3% hydrogen peroxide for 30 minutes. Subsequently, after the incubation with the corresponding primary antibody for 30 minutes, slides were washed and incubated for another 30 minutes with biotinylated universal secondary antibody followed by another incubation with HRP streptavidin (30 minutes) and diaminobenzidine for five minutes (kit LSAB+HRP Dako Cytomation Inc., Carpinteria, CA, USA) as a substrate. Slides were counterstained with hematoxylin for two minutes, dehydrated and mounted for conventional microscopy. Images of the stained lung parenchyma (tumor and non-tumor) were taken under a light microscope (Olympus, Series BX50F3, Olympus Optical Co., Hamburg, Germany) coupled with an image-digitizing camera (Pixera Studio, version 1.0.4, Pixera Corporation, Los Gatos, CA, USA). In addition, the number of ki-67 positively-stained nuclei was counted in the tumor and non-tumor lung specimens from a portion of the study patients (N=8). Data are expressed as the percentage of ki-67 positively-stained nuclei in each of the tumor and non-tumor specimens.

**Statistical analyses**

Statistical analyses were performed using the software statistical package for the social sciences (SPSS) 15.0 (SPSS Inc, Chicago, IL, USA). Data are expressed as mean (standard deviation). Normality of the variables was explored using the Shapiro-Wilk test. Variables detected in lung sample specimens from both groups of patients were analyzed independently using appropriate statistical approaches. For the quantitative variables in the lungs, differences between study groups were assessed using one-way analysis of variance (ANOVA) and Tukey’s *post-hoc* analysis to adjust for multiple comparisons of all the study variables. Chi-square test was used to assess potential differences between the study groups for the qualitative variables. Statistical significance was established at *P* ≤ 0.05.

**RESULTS**

**MicroRNA profile and downstream targets in tumors versus non-tumors specimens in both groups of study patients**

*E*xpression of miR-21 did not significantly differ between tumor and non-tumor lungs in LC or LC-COPD patients (Figure 1A). Expression of *PTEN* and *MARCKs* was significantly lower in tumor than non-tumor lungs only in LC-COPD patients (Table 2), while tumor expression of *TPM-1* was significantly greater in LC patients (Table 2). Expression of *PDCD4*, *SPRY-2*, miR-200b, *ETS-1* and *ZEB-2* did not differ between tumor and non-tumor lungs in any study group (Figure 1B and Table 2). In LC-COPD patients, miR-126 expression was significantly lower in tumors than non-tumor lungs (Figure 1C). Expression of *TOM-1* and *CRK* expression was significantly greater in tumors than non-tumor lungs only in LC patients, while an almost significant decrease in *EGFL-7* expression was observed (p=0.076) in LC-COPD patients (Table 2). Angiopoietin-2 protein levels were greater in tumor than non-tumor lungs in LC-COPD patients, whereas tumor fibulin-2 protein levels were increased in LC patients (Table 2 and Figure E1). Nevertheless, no significant differences were found in fibulin-3 or fibulin-5 levels between tumor and non-tumor lungs in any study group (Table 2 and Figure E1). Expression of miR-451 was significantly lower in tumor than non-tumor lungs only in LC-COPD patients (Figure 1D). In LC patients, *RAB-14* expression did not significantly differ between tumor and non-tumor lungs in any study group, whereas *MIF* expression significantly increased in tumors compared to non-tumor lungs (Table 2). Expression of miR-210 was significantly greater in tumors than non-tumor lungs in both LC and LC-COPD patients (Figure 2A). Expression of *FGFRL-1* was significantly lower in tumors than non-tumor lungs only in LC-COPD patients, while tumor *EFNA-3* expression was increased in LC patients (Table 2). In LC and LC-COPD patients, P62, LC3II/LC3I and BCL-2 levels significantly increased in tumors compared to non-tumor lungs, while beclin-1 and BAX protein levels were not modified in any study group (Table 2 and Figure E2),

Expression of miR-30a-30p was lower in tumors than non-tumors in both patient groups (Figure 2B). Expression of its downstream markers *SNAIL-1,* *P63*, and *CDKN1A* were lower in tumors than non-tumor lungs in LC-COPD patients, and in LC patients, tumor *CDKN1A* and *CDKN2A* expression levels were reduced (Table 2). No significant differences were found in *P53* expression between tumor and non-tumor lungs in the study groups (Table 2). Ki-67 positive-nuclei were significantly higher in tumors than non-tumor lungs of both study groups (Table 2 and Figure E3). Expression of miR-let7c was greater in tumor than non-tumor lungs in LC-COPD, whereas that of LC patients was reduced (Figure 2C). Expression of *k-RAS* was significantly lower in tumors than non-tumor lungs only in LC-COPD patients (Table 2). Expression levels of miR-155 and miR-let7a did not significantly differ between tumors and non-tumor lungs in any study group (Figures 2D and 2E).

**Differential profile of histone deacetylases and DNA methylation in lung tumors versus non-tumor specimens in both groups of study patients**

Expression of *SIRT-1* and HDAC2 did not differ between tumor and non-tumor lungs in any study group (Figures 3A, 3B, and E4). No significant differences were found in DNA methylation levels between tumor and non-tumor lungs in any study group (Figure 3C).

Reference List

(1) Miravitlles M. What was the impact of the Spanish COPD guidelines (GesEPOC) and how can they be improved? Arch Bronconeumol. 2016 Jan;52(1):1-2. doi: S0300-2896(15)00119-2 [pii];10.1016/j.arbres.2015.04.001 [doi].

(2) Miravitlles M, Soler-Cataluna JJ, Calle M, Molina J, Almagro P, Quintano JA, et al. Spanish Guidelines for Management of Chronic Obstructive Pulmonary Disease (GesEPOC) 2017. Pharmacological Treatment of Stable Phase. Arch Bronconeumol. 2017 Jun;53(6):324-35. doi: S0300-2896(17)30084-4 [pii];10.1016/j.arbres.2017.03.018 [doi].

(3) Vogelmeier CF, Criner GJ, Martinez FJ, Anzueto A, Barnes PJ, Bourbeau J, et al. Global Strategy for the Diagnosis, Management, and Prevention of Chronic Obstructive Lung Disease 2017 Report: GOLD Executive Summary. Arch Bronconeumol. 2017 Mar;53(3):128-49. doi: S0300-2896(17)30035-2 [pii];10.1016/j.arbres.2017.02.001 [doi].

(4) Mateu-Jimenez M, Curull V, Pijuan L, Sanchez-Font A, Rivera-Ramos H, Rodriguez-Fuster A, et al. Systemic and Tumor Th1 and Th2 Inflammatory Profile and Macrophages in Lung Cancer: Influence of Underlying Chronic Respiratory Disease. J Thorac Oncol. 2017 Feb;12(2):235-48. doi: S1556-0864(16)31177-7 [pii];10.1016/j.jtho.2016.09.137 [doi].

(5) Barreiro E, Fermoselle C, Mateu-Jimenez M, Sanchez-Font A, Pijuan L, Gea J, et al. Oxidative stress and inflammation in the normal airways and blood of patients with lung cancer and COPD. Free Radic Biol Med. 2013 Dec;65:859-71. doi: S0891-5849(13)00407-3 [pii];10.1016/j.freeradbiomed.2013.08.006 [doi].

(6) Mateu-Jimenez M, Sanchez-Font A, Rodriguez-Fuster A, Aguilo R, Pijuan L, Fermoselle C, et al. REDOX IMBALANCE IN LUNG CANCER OF PATIENTS WITH UNDERLYING CHRONIC RESPIRATORY CONDITIONS. Mol Med. 2016 Jan 7; doi: molmed.2015.00199 [pii];10.2119/molmed.2015.00199 [doi]. Pubmed PMID: PMC5004710.

(7) Puig-Vilanova E, Ausin P, Martinez-Llorens J, Gea J, Barreiro E. Do epigenetic events take place in the vastus lateralis of patients with mild chronic obstructive pulmonary disease? PLoS One. 2014;9(7):e102296. doi: 10.1371/journal.pone.0102296 [doi];PONE-D-14-13450 [pii].

(8) Puig-Vilanova E, Rodriguez DA, Lloreta J, Ausin P, Pascual-Guardia S, Broquetas J, et al. Oxidative stress, redox signaling pathways, and autophagy in cachectic muscles of male patients with advanced COPD and lung cancer. Free Radic Biol Med. 2015 Feb;79:91-108. doi: S0891-5849(14)01369-0 [pii];10.1016/j.freeradbiomed.2014.11.006 [doi].

(9) Puig-Vilanova E, Martinez-Llorens J, Ausin P, Roca J, Gea J, Barreiro E. Quadriceps muscle weakness and atrophy are associated with a differential epigenetic profile in advanced COPD. Clin Sci (Lond). 2015 Jun;128(12):905-21. doi: CS20140428 [pii];10.1042/CS20140428 [doi].

(10) Livak KJ, Schmittgen TD. Analysis of relative gene expression data using real-time quantitative PCR and the 2(-Delta Delta C(T)) Method. Methods. 2001 Dec;25(4):402-8. doi: 10.1006/meth.2001.1262 [doi];S1046-2023(01)91262-9 [pii].

(11) Chacon-Cabrera A, Gea J, Barreiro E. Short- and Long-Term Hindlimb Immobilization and Reloading: Profile of Epigenetic Events in Gastrocnemius. J Cell Physiol. 2016 Oct 7; doi: 10.1002/jcp.25635 [doi].

(12) Puig-Vilanova E, Aguilo R, Rodriguez-Fuster A, Martinez-Llorens J, Gea J, Barreiro E. Epigenetic mechanisms in respiratory muscle dysfunction of patients with chronic obstructive pulmonary disease. PLoS One. 2014;9(11):e111514. doi: 10.1371/journal.pone.0111514 [doi];PONE-D-14-19769 [pii]. Pubmed PMID: PMC4219759.

(13) Mateu-Jimenez M, Fermoselle C, Rojo F, Mateu J, Pena R, Urtreger AJ, et al. Pharmacological Approaches in an Experimental Model of Non-Small Cell Lung Cancer: Effects on Tumor Biology. Curr Pharm Des. 2016;22(34):5300-10. doi: CPD-EPUB-76751 [pii].

**Table S1. Description of the microRNAs analyzed in the study**

| **Assay Name** | **Assay ID** | **miRBase accession number** |
| --- | --- | --- |
| hsa-let7c | 000379 | MIMAT0000064 |
| hsa-miR-21 | 000397 | MIMAT0013029 |
| hsa-miR-30a-3p | 000416 | MIMAT0006914 |
| hsa-miR-126 | 000451 | MIMAT0026206 |
| hsa-miR-210 | 000512 | MIMAT0023849 |
| hsa-miR-451 | 001141 | MIMAT0001634 |
| hsa-miR-200b | 00225 | MIMAT0021847 |
| hsa-miR155 | 002287 | MIMAT0004658 |
| hsa-let7a | 00377 | MIMAT0000062 |
|  |  | **NCBI Accession number** |
| U6 snRNA, housekeeping gene | 001973 | NR_004394 |

*Abbreviations*: ID, identification; hsa, homo sapiens; miR, microRNA; MIMAT, mature microRNA; snRNA, small nuclear RNA; and NR, non-coding RNA RefSeq database category.

**Table S2. Target genes analyzed in the study.**

|  | **Assay ID** | **GeneBank accession number** |
| --- | --- | --- |
| PTEN | Hs02621230_s1 | NM_000314.4 |
| CRK | Hs00180418_m1 | NM_016823.3 |
| HRAS | Hs00610483_m1 | NM_001130442.1 |
| PDCD4 | Hs00377253_m1 | NM_145341.3 |
| TOM1 | Hs00193953_m1 | NM_001135729.1 |
| TPM1 | Hs00165966_m1 | NM_001018004.1 |
| MIF | Hs00236988_g1 | NM_002415.1 |
| SPRY2 | Hs00183386_m1 | NM_005842.2 |
| RAB14 | Hs00249440_m1 | NM_016322.3 |
| MARCKS | Hs00158993_m1 | NM_002356.5 |
| FGFRL1 | Hs00222484_m1 | NM_001004356.2 |
| ETS1 | Hs00428293_m1 | NM_001143820.1 |
| HOXA1 | Hs00939046_m1 | NM_153620.2 |
| ZEB2 | Hs00207691_m1 | NM_001171653.1 |
| EFNA3 | Hs00191913_m1 | NM_004952.4 |
| EGFL7 | Hs00211952_m1 | NM_201446.2 |
| SNAIL1 | Hs00195591_m1 | NM_005985.3 |
| TP53 | Hs01034249_m1 | NM_001126112.2 |
| TP63 | Hs00978343_m1 | NM_001114978.1 |
| CDKN1A | Hs00355782_m1 | NM_001220778.1 |
| CDKN2A | Hs00923894_m1 | NM_058197.4 |
| SIRT1 | Hs01009006_m1 | NM_001142498.1 |
| GAPDH | Hs99999905_m1 | NM_002046.4 |

**FIGURE LEGENDS**

**Figure S1:** Representative immunoblots of fibulin-3, fibulin-5, fibulin-2, ANG-2 and GAPDH in the lung tumor (T) and non-tumor (NT) of LC and LC-COPD patients. Definition of abbreviations: Mw, molecular weight; LC, lung cancer; COPD, chronic obstructive pulmonary disease; ANG-2, angiopoietin-2; and GAPDH, [glyceraldehyde 3-phosphate dehydrogenase](https://en.wikipedia.org/wiki/Glyceraldehyde_3-phosphate_dehydrogenase).

**Figure S2:** Representative immunoblots of P-62, Beclin-1, LC3II/I, BAX, BCL-2 and GAPDH in the lung tumor (T) and non-tumor (NT) of LC and LC-COPD patients. Definition of abbreviations: Mw, molecular weight; LC, lung cancer; COPD, chronic obstructive pulmonary disease; LC3, light-chain 3; BAX, bcl-2 associated x protein; BCL-2, b-cell lymphoma 2; and GAPDH, [glyceraldehyde 3-phosphate dehydrogenase](https://en.wikipedia.org/wiki/Glyceraldehyde_3-phosphate_dehydrogenase).

**Figure S3:** Representative examples of ki-67 immunohistochemical staining in non-tumor (panel A) and tumor (panel B) lung specimens.

**Figure S4:** Representative immunoblots of HDAC2 and GAPDH in the lung tumor (T) and non-tumor (NT) of LC and LC-COPD patients. Definition of abbreviations: Mw, molecular weight; LC, lung cancer; COPD, chronic obstructive pulmonary disease; HDAC2, histone deacetylase 2; and GAPDH, [glyceraldehyde 3-phosphate dehydrogenase](https://en.wikipedia.org/wiki/Glyceraldehyde_3-phosphate_dehydrogenase).


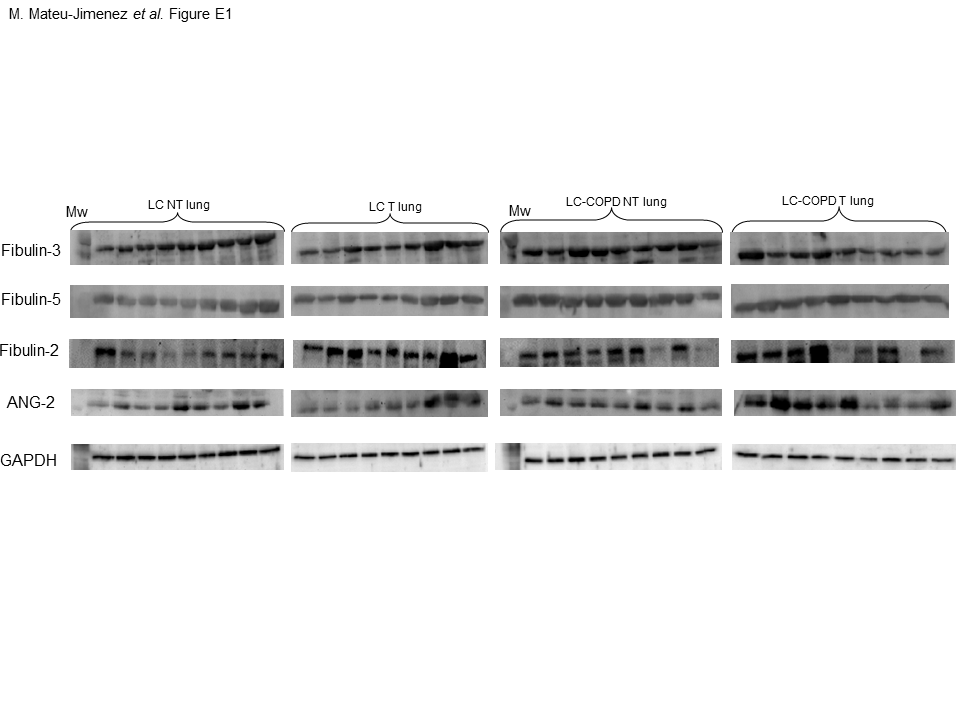


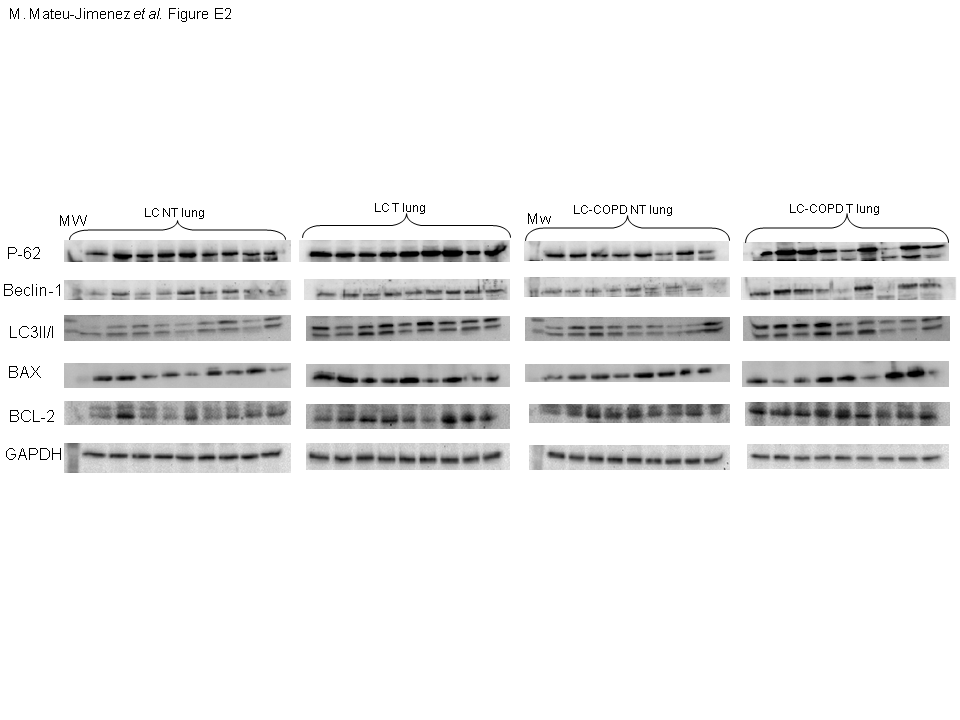


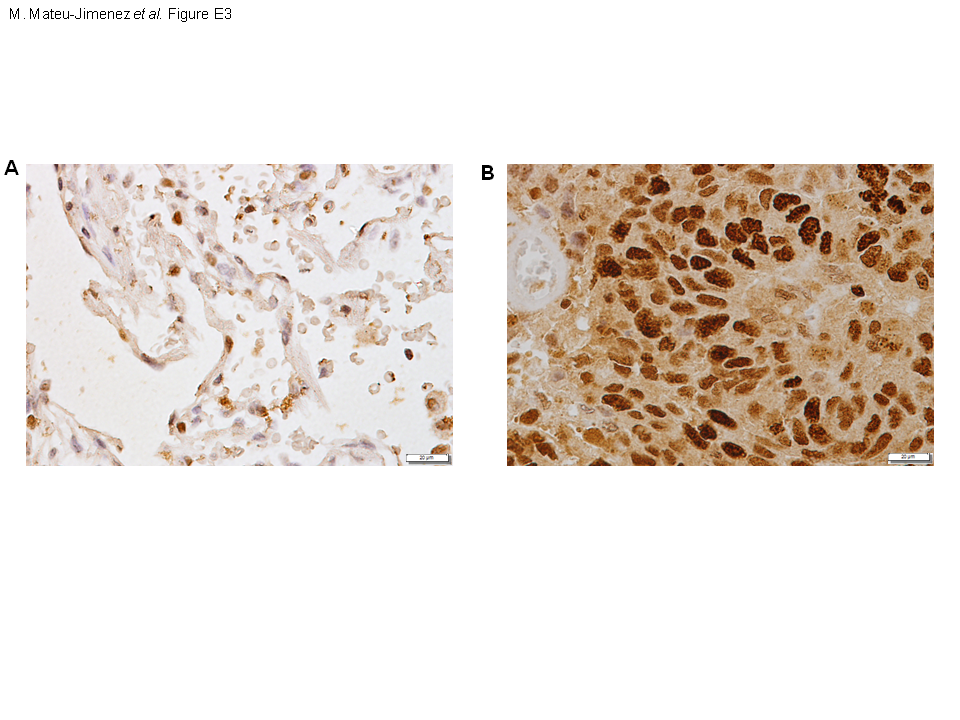


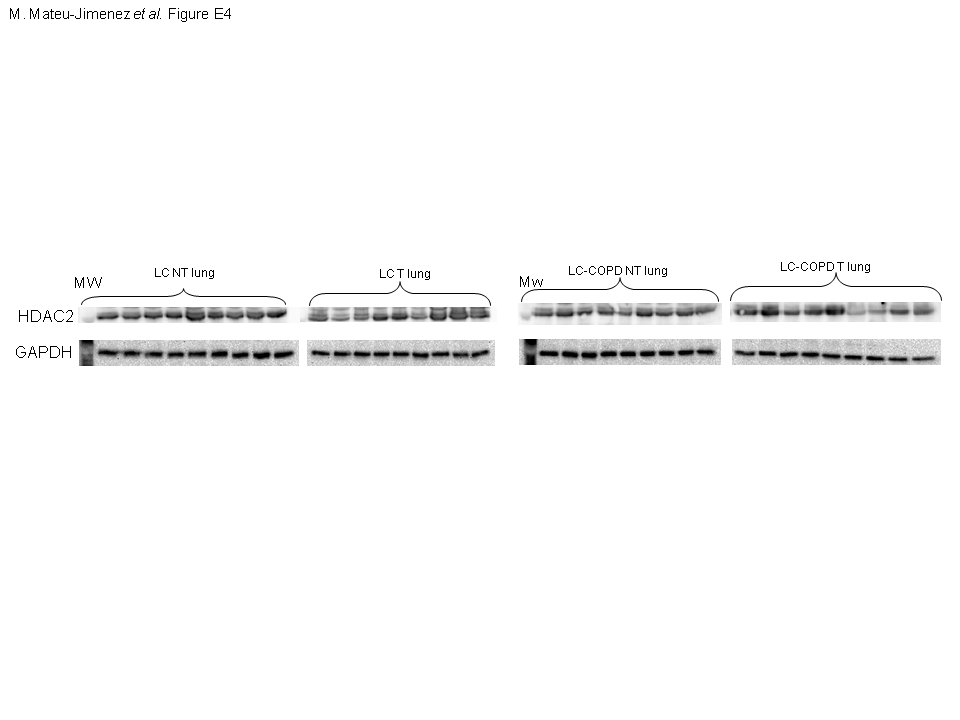

Supplement: Additional file 1: — Detailed information on the methodologies used in the study as well as the immunoblot images of the analysed markers. (DOCX 1563 kb) [file 13148_2017_437_MOESM1_ESM.docx]
